# Correlates of exposure to secondhand smoke (SHS) at home among non-smoking adults in Bangladesh: findings from the ITC Bangladesh survey

**DOI:** 10.1186/1471-2466-14-117

**Published:** 2014-07-16

**Authors:** Abu S Abdullah, Pete Driezen, Genevieve Sansone, Nigar Nargis, Ghulam AKM Hussain, Anne CK Quah, Geoffrey T Fong

**Affiliations:** 1 School of Public Health, Guangxi Medical University, 22 Shuangyong Road, Nanning, Guangxi 530021, China; 2 Boston University School of Medicine, Boston Medical Center, Boston, Massachusetts, USA; 3 Propel Centre for Population Health Impact, University of Waterloo, Waterloo, Ontario, Canada; 4 Department of Economics, University of Dhaka, Dhaka, Bangladesh; 5 Department of Psychology, University of Waterloo, Waterloo, Ontario, Canada; 6 Ontario Institute for Cancer Research, Toronto, Ontario, Canada

**Keywords:** Secondhand smoke (SHS), Tobacco smoke pollution (TSP), Bangladesh, Home, Non-smoker

## Abstract

**Background:**

Exposure to secondhand smoke (SHS) is a serious global public health problem. Understanding the correlates of SHS exposure could guide the development of evidence based SHS exposure reduction interventions. The purpose of this study is to describe the pattern of and factors associated with SHS exposure among non-smoking adults in Bangladesh.

**Methods:**

Data come from adult non-smokers who participated in the second wave (2010) of the International Tobacco Control Policy (ITC) Evaluation Bangladesh Survey conducted in all six administrative divisions of Bangladesh. A structured questionnaire gathered information on participants’ demographic characteristics, pattern of SHS exposure, SHS knowledge, and attitudes towards tobacco control. Exposure to SHS at home was defined as non-smokers who lived with at least one smoker in their household and who reported having no home smoking ban. The data were analyzed using chi-square tests and logistic regression procedures.

**Results:**

The SHS exposure rate at home among the participants (N = 2813) was 43%. Several sociodemographic and attitudinal factors were associated with SHS exposure. Logistic regression analyses identified eight predictors of SHS exposure: being female (OR = 2.35), being aged 15–24 (OR = 2.17), being recruited from Dhaka slums (OR = 5.19) or non-tribal/non-border areas outside Dhaka (OR = 2.19) or tribal/border area (OR = 4.36), having lower education (1–8 years: OR = 2.45; illiterate: OR = 3.00, having higher monthly household income (5000 to <10,000 Taka: OR = 2.34; 10,000 Taka or more: OR = 2.28), having a father who smoked in the past or currently smokes (OR = 2.09), having lower concern about the harms of tobacco on children (unconcerned OR = 3.99; moderate concern OR = 2.26), and not knowing the fact that SHS causes lung cancer in non-smokers (OR = 2.04).

**Conclusions:**

Almost half of non-smoking Bangladeshi adults are exposed to SHS at home. The findings suggest the need for comprehensive tobacco control measures that would improve public understanding about health hazards of SHS exposure at home and encourage educational initiatives to promote smoke-free homes. Interventions should deliver targeted messages to reach those in the low socioeconomic status group.

## Background

Secondhand smoke (SHS), the smoke generated by active smokers, remains a widespread health hazard worldwide [1,2]. Several studies have clearly linked exposure to SHS, also known as tobacco smoke pollution (TSP) or passive smoking, to a number of health consequences in non-smokers, including lung cancer, heart disease, and asthma in children [1-3]. The World Health Organization (WHO) has estimated that in 2004, about one-third of adults and 40% of children worldwide were exposed to SHS, and that this exposure caused 1.0% of all deaths and 0.7% of the worldwide burden of disease and disability-adjusted life years in that year [4]. Smoke-free policies for public and private environments are the most effective way to reduce SHS exposure, and therefore decrease the SHS induced health consequences [1].

In response to the growing awareness of the health risks posed by SHS, an increasing number of countries have introduced legislation to reduce or eliminate exposure to SHS in public places. Article 8 of the WHO Framework Convention on Tobacco Control (FCTC) includes guidelines for protection from TSP [5]. These guidelines recommend comprehensive bans on smoking in public places and workplaces in order to achieve 100% smoke-free environments. Households, however, are not protected under the FCTC, thus household smoking bans must be initiated voluntarily. As a result, even as smoke-free restrictions in public places are becoming more widespread, the home remains a predominant source of exposure to SHS, particularly among vulnerable children [1,4].

During the last decade, a significant amount of research had been conducted addressing the SHS exposure at home. However, the majority of research on household exposure to SHS comes from developed or high income countries, and information about the prevalence of SHS exposure in the home and measures to reduce this exposure in developing countries is limited.

It was argued that the actual conditions of SHS exposure and the burden of the resulting health outcomes differ greatly between developed and developing countries [6,7]. For example, factors such as the number of smokers in the home, the intensity of the smoke, construction aspects of the home (i.e. space, ventilation), and even cultural factors such as gender dynamics can all influence the experience of and the impact of SHS exposure in the home among non-smokers and children in developing countries. Information on the pattern and correlates of SHS exposure at home in developing country would guide the development of evidence-based educational and policy initiative, specific to developing countries.

With 41.1 million tobacco users in Bangladesh, including 20.9 million people who smoke, [8] exposure to SHS at home is a major public health concern. In Bangladesh, smoking is prohibited in public places and public transportation; however, designated smoking areas are permitted [9]. Data on SHS exposure in Bangladesh is limited; however some information on the prevalence of SHS exposure is available from the 2009 Global Adult Tobacco Survey (GATS) [10]. According to the GATS Bangladesh survey, 63% and 45% of all adult workers were exposed to tobacco smoke at the workplace and in public places, respectively, and 76% and 70% of non-smokers were exposed in these places [10]. In the absence of any data, it could be estimated that a significant proportion of Bangladeshi non-smokers and children are exposed to SHS at home. A 2011 study conducted among Bangladeshi households with children [11] reported a high prevalence (67%) of SHS exposure among children. In this study, only 43.5% of respondents had complete smoking restrictions in the home. This low prevalence of home smoking restrictions suggests that many non-smokers who live with at least one smoker in the home are regularly exposed to SHS.

The objective of this study was to examine the patterns of SHS exposure at home and its demographic and behavioural correlates in non-smoking adults in Bangladesh. Furthering understanding of the patterns of SHS exposure at home among adult non-smokers and identification of factors that are associated with SHS exposure would guide the development of SHS exposure reduction intervention strategies at home.

## Methods

### Data source

The data for this study come from Wave 2 of the International Tobacco Control (ITC) Policy Evaluation Bangladesh Survey. A detailed description of the ITC Bangladesh Survey can be found elsewhere [11,12]. Briefly, the ITC Bangladesh Survey is a prospective cohort survey of a nationally representative sample of smokers and non-smokers conducted in all six administrative divisions of Bangladesh: Barisal, Chittagong, Dhaka, Khulna, Rajshahi, and Sylhet. The target population of the ITC Bangladesh Survey consists of adult users and non-users of tobacco ages 15 years and older. In Wave 1, we surveyed 3111 smokers and 2660 non-smokers during March and June, 2009. Of these, 2521 smokers and 2100 non-smokers were successfully followed to Wave 2 in 2010. Fifty-eight of the Wave 1 non-smokers had started smoking by Wave 2. Another 622 smokers and 553 non-smokers were recruited in Wave 2 to replace those respondents lost to attrition. These respondents were recruited using the same sampling design as in Wave 1. Most of these new recruits (78%) were from the slum areas of Dhaka where attrition was greatest. The sample for this study is based on 2813 non-smoking respondents participating in Wave 2, of whom 219 were smokers in Wave 1 but had quit by Wave 2, 2041 were non-smokers in Wave 1 and followed successfully to Wave 2 and 553 were newly recruited non-smokers in Wave 2. All data were collected using face-to-face interviews. Sampling weights were also computed so that results are representative of the Bangladeshi adult population.

In all waves of the ITC Bangladesh surveys, written consent was obtained from those participants who can read and write; others gave verbal consent. Ethical approval was obtained from the Office of Research Ethics at the University of Waterloo (Waterloo, Canada), and the Ethical Review Committee, Bangladesh Medical Research Council.

### Measures

A standardized Bengali questionnaire was used for data collection. Details of the questionnaire were described elsewhere [11]. Briefly, the questionnaire obtained information on the subject’s *socio-demographic background* (sex, age, religion, residence (rural, urban, slum), marital status, educational attainment, occupation, total household monthly income), *smoking behavior*, *use of any smokeless tobacco*, and *exposure to SHS*. Non-smokers who were *exposed to SHS* were those who lived with at least one smoker in their household and who reported having no home smoking ban. If the non-smoking respondent was the only person interviewed, exposure to SHS is based only on this respondent’s report of home smoking bans. If this respondent reported that smoking is “Not allowed at all” then this person was classified as “not exposed” to second-hand smoke. If this person reports that smoking is allowed in some or all areas of the home, then this person was classified as exposed to SHS (or at least, potentially exposed).

If more than one respondent was interviewed in the home (>1 non-smoker or, more commonly, 1 non-smoker and 1 smoker), then exposure status is based on all reports of home smoking bans. If all interviewed respondents report that smoking is “Not allowed at all” then the interviewed non-smoker was classified as “not exposed” to SHS. If any of the reports conflicted, so that at least one person reports that smoking is allowed in some or all areas of the home, then the non-smoker was classified as “exposed” to SHS. *Knowledge of the health consequences of SHS exposure* was also assessed, along with opinions towards smoking restrictions. To measure knowledge of the health consequences of SHS exposure, respondents were asked: “Based on what you know or believe, does passive smoking cause…?”, followed by a list of health effects. Measures from the list included in the present study were: lung cancer in non-smokers, and asthma in children. To assess opinions on suggestions from friends and families, respondents were asked: “do you talk to friends before making a decision” or “will you give up smoking if family disapproves”?” To measure opinions on smoking restrictions, respondents were asked: “For each of the following public places, please tell me if you think smoking should not be allowed in any indoor areas, should be allowed only in some indoor areas, or no rules or restrictions?” The list included: hospitals, workplaces, restaurants or tea stalls, public transportation vehicles, and schools/colleges/universities.

### Statistical analysis

Descriptive statistics appropriate for complex survey data were used to estimate the prevalence of exposure to SHS among non-smokers living in Bangladesh. Associations between tobacco smoke pollution and (i) demographics, (ii) tobacco use behaviours and (ii) knowledge and opinions of SHS were tested using the Rao-Scott χ^2^ test. Variables that were significantly correlated with SHS exposure (p < 0.05) were entered into a stepwise logistic regression model to identify predictors of SHS exposure using p < 0.05 as a criterion to enter and remove variables from the model. The final selected model was re-fit using a logistic regression model appropriate for complex survey data that accounts for the complex design and incorporated the sampling weights. In all logistic regression models, variables used in the construction of the sampling weights (sex, age and residential location) were forced into the model to reduce biases in the other coefficients. Unless otherwise indicated, all results were weighted using the sampling weights. To elaborate, the computation of sampling weights began with a village level household weight for all households enumerated within the sampled villages. From this weight, a national level household weight was computed as the approximate number of households, at the national level, represented by an enumerated household. Then, for each household where an interviewed was conducted, a national level interview household weight was computed. Then, for each interviewed individual, an individual level weight within household was computed. The product of the interviewed household weight and individual within-household weight was calibrated to sum to population estimates in groups defined by geography and demographics. Final weights were rescaled to national sample sizes. The analysis also accounted for the multi-stage sampling design employed in the ITC Bangladesh Survey using the complex samples survey routines available in SAS Version 9.3.

## Results

Table 1 shows the sociodemographic characteristics of the sample. Of the non-smoking respondents (N = 2813), three quarters (74%) were female and the highest percentage of respondents (38%) were 25 to 39 years of age. The vast majority of respondents were married (80%) and Muslim (84%). About one-half of respondents lived in rural areas (54%), had a moderate (1 to 8 years) level of formal education (54%) and were in the middle category for monthly household income (between 5,000 and 10,000 taka; 46%). In addition, three quarters (75%) of the respondents had a family member who smoked in the past or currently smokes and 22% of the respondents were current smokeless tobacco users.

**Table 1:** Characteristics of non-smokers participating in Wave 2 of the ITC Bangladesh Survey (unweighted, n = 2813)

	**Freq.**	**(%)**
**Wave of recruitment**		
1	2260	(80.3)
2	553	(19.7)
**Recruitment area**		
Dhaka (non-slum)	271	(9.6)
Dhaka slums	541	(19.2)
Non-tribal, non-border areas outside Dhaka	1893	(67.3)
Tribal/border areas	108	(3.8)
**Residence**		
Urban (non-slum areas)	745	(26.5)
Urban slums	541	(19.2)
Rural	1527	(54.3)
**Sex**		
Male	731	(26.0)
Female	2082	(74.0)
**Age group**		
15-24	781	(27.8)
25-39	1079	(38.4)
40-54	609	(21.6)
55+	344	(12.2)
**Marital status**		
Married	2243	(79.7)
Otherwise	570	(20.3)
**Religion**		
Muslim	2356	(84.1)
Otherwise	446	(15.9)
**Education**		
Illiterate	708	(25.2)
1-8 years	1505	(53.6)
9+ years	597	(21.2)
**Monthly household income**		
< 5,000 taka	498	(17.7)
5,000 to < 10,000 taka	1297	(46.1)
> = 10,000 taka	801	(28.5)
Not reported	217	(7.7)
**Has microfinance loan**		
Does not have a loan	2330	(82.9)
Has a loan	481	(17.1)
**Self-rated health**		
Poor	109	(3.9)
Average	886	(31.6)
Good	1546	(55.2)
Excellent	262	(9.3)
**Age of youngest child in the home**		
No children	743	(26.4)
Youngest < = 5	1356	(48.2)
Youngest 6 to 13	714	(25.4)
**Father smoked/currently smokes tobacco**		
Never smoked	708	(26.3)
Was a smoker/currently smokes	1981	(73.7)
Anyone* in family smoked/currently smokes tobacco		
Never smoked	711	(25.5)
Was a smoker/currently smokes	2081	(74.5)
**Currently uses smokeless tobacco**		
Non-user	2193	(78.4)
Smokeless user	605	(21.6)
**Tobacco status**		
Recent quitter	219	(7.8)
Non-smoker	2594	(92.2)

*Father/mother/grandfather/grandmother.

Overall, 43% (95% CI: 36.9% – 48.5%) of Bangladeshi non-smokers are exposed to SHS. Table 2 shows that exposure to SHS varied by important demographic characteristics. Exposure to SHS was lowest among residents of Dhaka city (34%) and highest among residents of the Dhaka slums (64%). A greater percentage of women were exposed to SHS (48%) than men (34%). Younger adults were more likely to be exposed to SHS than older adults (p < 0.001). Illiterate Bangladeshis were also more likely to be exposed to SHS than the most educated (52% vs. 29%, respectively). Exposure also varied by household income (those in the highest income category were less likely to be exposed, p <0.001) and whether they had microfinance loan (those who had a loan were more likely to be exposed, p <0.02). Furthermore, non-smokers whose father smoked were more likely to be exposed to SHS (48%) than those whose father did not smoke (31%) (p <0.001). Also, SHS exposure was higher among those who had a smoking family member (47%) than those who did not have one (31%) (p <0.001). Exposure did not significantly differ by urban/rural area, marital status, religion, self-rated health, age of the youngest child in the home, or smokeless tobacco use.

**Table 2:** Prevalence of exposure to second-hand smoke (weighted)

	**Unwtd**	**Freq**	**%**	**(SE)**	**(95% CI)**	**Rao-Scott ChiSq**	**DF**	**p value**
**Overall prevalence**								
Exposed to SHS	1635/	2813	42.6	(2.88)	(36.9,48.5)	Not applicable		
**Area**								
Dhaka (non-slum)	121/	271	33.8	(1.14)	(31.6,36.2)	15.01	3	0.002
Dhaka slums	348/	541	64.0	(8.87)	(44.9,79.5)				
Non-tribal, non-border areas outside Dhaka	1108/	1893	42.4	(3.16)	(36.2,48.9)				
Tribal/border areas	58/	108	49.3	(2.64)	(44.0,54.6)				
**Residence**									
Urban (non-slum areas)	373/	745	38.4	(5.07)	(28.8,49.1)	4.07	2	0.130	
Urban slums	348/	541	64.0	(8.87)	(44.9,79.5)				
Rural	914/	1527	43.8	(3.00)	(37.9,49.9)				
**Sex**									
Male	330/	731	34.1	(3.95)	(26.6,42.5)	12.98	1	< 0.001	
Female	1305/	2082	47.7	(2.93)	(41.8,53.6)				
**Age group**									
15-24	500/	781	51.3	(4.34)	(42.5,59.9)	11.40	3	0.010	
25-39	608/	1079	39.7	(4.03)	(31.9,48.1)				
40-54	345/	609	38.1	(2.94)	(32.3,44.2)				
55+	182/	344	39.1	(4.66)	(30.2,48.8)				
**Marital status**									
Married	1312/	2243	41.0	(3.10)	(35.8,48.3)	0.28	1	0.594	
Otherwise	323/	570	44.4	(4.49)	(35.6,53.6)				
**Religion**									
Muslim	1394/	2356	42.8	(2.91)	(37.0,48.7)	0.09	1	0.762	
Otherwise	233/	446	40.9	(6.09)	(29.4,53.6)				
**Education**									
Illiterate	459/	708	52.3	(4.49)	(43.3,61.3)	19.75	2	< 0.001	
1-8 years	916/	1505	45.7	(3.37)	(39.0,52.5)				
9+ years	258/	597	29.2	(4.22)	(21.5,38.4)				
**Monthly household income**									
< 5,000 taka	309/	498	44.6	(4.95)	(34.9,54.7)	15.82	3	0.001	
5,000 to < 10,000 taka	806/	1297	49.2	(3.34)	(42.5,56.0)				
≥ 10,000 taka	441/	801	39.5	(3.90)	(31.9,47.6)				
Not reported	79/	217	25.9	(5.70)	(16.1,38.9)				
**Has microfinance loan**									
Does not have microfinance loan	1327/	2330	40.3	(2.71)	(35.0,45.9)	5.97	1	0.015	
Has microfinance loan	307/	481	53.1	(5.92)	(41.2,64.7)				
**Self-rated health**									
Poor	68/	109	32.7	(8.28)	(18.5,51.0)	1.46	3	0.691	
Average	495/	886	41.9	(3.37)	(35.3,48.9)				
Good	910/	1546	43.9	(3.70)	(36.6,51.4)				
Excellent	154/	262	41.9	(6.69)	(29.3,55.8)				
**Age of youngest child in the home**									
No children	408/	743	44.2	(3.70)	(36.9,51.8)	0.43	2	0.807	
Youngest ≤ 5	806/	1356	42.3	(3.58)	(35.3,49.7)				
Youngest 6 to 13	421/	714	41.5	(3.73)	(34.2,49.2)				
**Father smoked/currently smokes tobacco**									
Never smoked	352/	708	31.1	(2.38)	(26.5,36.1)	14.00	1	< 0.001	
Was a smoker/currently smokes	1205/	1981	47.5	(3.91)	(39.7,55.4)				
Anyone* in family smoked/currently smokes									
No one smoked	359/	711	31.3	(2.31)	(26.8,36.1)	15.37	1	< 0.001	
Someone was/is a smoker	1262/	2081	46.9	(3.72)	(39.5,54.4)				
**Currently uses smokeless tobacco**									
Non-user	1274/	2193	42.1	(3.04)	(36.1,48.3)	0.09	1	0.764	
Smokeless user	351/	605	43.4	(4.57)	(34.5,52.8)				

*Father/mother/grandfather/grandmother.

As shown in Table 3, few of the attitudinal/knowledge measures were related to SHS exposure. Non-smokers who agreed (48%) or strongly agreed (42%) that smoked tobacco is addictive were significantly less likely to be exposed to SHS than those who did not agree (64%) (p < 0.05). Non-smokers who were very/extremely concerned about the harms of tobacco smoke to children were significantly less likely to be exposed to SHS (43%) than either those who were moderately concerned (61%) or unconcerned (74%) (p <0.001). Those who believed that SHS causes lung cancer in non-smokers were significantly less likely to be exposed to SHS (40%) than those who did not know this fact (62%) (p <0.001). Non-smokers who believed that SHS causes asthma in children were significantly less likely to be exposed to SHS (41%) than either those who did not believe (50%) or did not know (56%) this fact (p <0.001).

**Table 3:** Attitudinal and knowledge related predictors of SHS exposure (weighted)

	**Exposure to SHS**						
		**Not exposed**		**Exposed**			
	**Freq.**	**%**	**Freq.**	**%**	**Rao-Scott ChiSq**	**DF**	**p value**
**Smoking should be completely banned in hospitals**							
Complete ban	1135	53.7	1578	46.3	0.11	1	0.739
Otherwise	41	58.6	38	41.4			
**Smoking should be completely banned in workplaces**							
Complete ban	988	54.2	1303	45.8	0.96	1	0.327
Otherwise	157	57.3	222	42.7			
**Smoking should be completely banned in restaurants**							
Complete ban	946	55.0	1237	45.0	0.22	1	0.636
Otherwise	193	53.2	273	46.8			
**Smoking should be completely banned in public transit**							
Complete ban	1120	53.8	1546	46.2	0.03	1	0.862
Otherwise	49	56.8	55	43.2			
**Smoking should be completely banned in schools**							
Complete ban	1086	53.2	1512	46.8	0.16	1	0.686
Otherwise	51	59.6	46	40.4			
**Smoked tobacco is addictive**							
Does not agree	40	35.6	80	64.4	6.40	2	0.041
Agree	577	52.3	822	47.7			
Strongly agree	542	57.8	697	42.2			
**Society disapproves of smoking**							
Does not agree	95	49.4	162	50.6	0.80	2	0.669
Agree	684	55.6	892	44.4			
Strongly agree	371	57.6	456	42.4			
**Have smoking restrictions at work**							
Not employed	926	51.6	1381	48.4	5.86	2	0.053
Employed, no restrictions	156	60.6	171	39.4			
Employed, some/complete restrictions	96	59.7	83	40.3			
**Talk to friends before making a decision**							
Does not agree	176	44.1	296	55.9	3.96	2	0.138
Agree	734	56.6	902	43.4			
Strongly agree	242	54.9	366	45.1			
**Will give up activities if family disapproves**							
Does not agree	118	49.3	194	50.7	0.57	2	0.750
Agree	706	54.3	926	45.7			
Strongly agree	221	58.2	260	41.8			
**Concern that tobacco smoke harms children**							
Very/extremely concerned	804	57.3	937	42.7	27.84	3	< 0.001
No children in the home	125	60.0	155	40.0			
Unconcerned/a little concerned	14	25.9	47	74.1			
Moderate concerned	170	39.4	408	60.6			
**Second-hand smoke causes lung cancer in non-smokers**							
Yes	918	60.3	994	39.7	20.14	2	< 0.001
No	52	59.6	43	40.4			
Don’t know	195	37.6	569	62.4			
**Second-hand smoke causes asthma in kids**							
Yes	867	58.7	960	41.3	7.11	2	0.029
No	36	50.0	39	50.0			
Don’t know	259	43.8	603	56.2			

The stepwise logistic regression model (unweighted) identified eight predictors of SHS exposure: sex, age, recruitment area (Dhaka non-slum, Dhaka slum, non-tribal/non-border, tribal/border), education, income, whether father smoked, concern that tobacco smoke harms children and believing that SHS causes lung cancer in non-smokers. This model was re-estimated using a complex samples logistic regression model to estimate the effect of each predictor on SHS exposure (Table 4). Based on this model, women had 2 times greater odds of being exposed to SHS than men. The youngest Bangladeshis (aged 15–24) had 2 times greater odds of being exposed to SHS than the oldest Bangladeshis (aged 55 and older). Social gradients in exposure were also found, with illiterate and less educated Bangladeshis having higher odds of exposure compared to the more highly educated. However, Bangladeshis with a higher income (taka 5000 or more) had greater odds of being exposed to SHS compared to low income (<5000 taka) Bangladeshis. Residents other than the Dhaka non-slum areas had higher odds of SHS exposure. Bangladeshis who did not know that SHS causes lung cancer had 2 times greater odds of being exposed to SHS than those who knew this fact. Finally, concern about smoking in the presence of children was significantly associated with the odds of SHS exposure even *after* controlling for all other predictors: those Bangladeshis who were unconcerned/a little concerned had the 3.45 times the odds of exposure compared to those who were very/extremely concerned.

**Table 4:** Odds of being exposed to second-hand smoke (weighted; n = 2472)

			**Wald ChiSq test**		
	**Odds ratio**	**(95% CI)**	ChiSq*	**df**	**p value**
**Sex**					
Male	1.00	--	16.12	1	< 0.001
Female	2.35	(1.55, 3.56)			
**Age group**					
55+	1.00	--	19.81	3	< 0.001
40-54	0.91	(0.61, 1.34)			
25-39	0.97	(0.56, 1.69)			
15-24	2.17	(1.26, 3.75)			
**Area**					
Dhaka non-slum areas	1.00	--	20.85	3	< 0.001
Dhaka slums	5.19	(1.92, 14.05)			
Non-tribal/non-border areas outside Dhaka	2.19	(1.38, 3.48)			
Tribal/border areas	4.36	(1.89, 10.07)			
**Tobacco use status**					
Non-smoker	1.00	--	3.01	1	0.083
Former smoker	1.50	(0.95, 2.37)			
**Education**					
9 years or more	1.00	--	32.76	2	< 0.001
1- 8 years	2.45	(1.80, 3.33)			
Illiterate	3.00	(1.91, 4.71)			
**Income**					
< 5,000 taka	1.00	--	32.24	3	< 0.001
5,000 to < 10,000 taka	2.34	(1.59, 3.43)			
10,000 taka or more	2.28	(1.54, 3.38)			
Not reported	0.62	(0.32, 1.19)			
**Father smoked/currently smokes**					
Father never smoked	1.00	--	15.92	1	< 0.001
Father used to/currently smokes	2.09	(1.45, 3.00)			
**Concern that tobacco smoke harms children**					
Very/extremely concerned	1.00	--	28.63	3	< 0.001
No children in the home	0.87	(0.61, 1.23)			
Unconcerned/a little concerned	3.45	(1.03, 11.51)			
Moderately concerned	1.95	(1.35, 2.83)			
**Second-hand smoke causes lung cancer in non-smokers**					
Yes	1.00	--	15.05	2	< 0.001
No	0.70	(0.27, 1.81)			
Don’t know	2.04	(1.17, 3.56)			
**Second-hand smoke causes asthma in children**					
Yes	1.00	--	1.87	2	0.393
No	1.23	(0.31, 4.89)			
Don’t know	0.70	(0.41, 1.20)			

*Omnibus test.

## Discussion

The findings of this study show that almost half (43%; 95% CI: 37%-49%) of non-smoking Bangladeshi adults are exposed to SHS at home. To our knowledge this is the first study to report SHS exposure in the home among non-smoking adults in Bangladesh. The SHS exposure rate in the current study is lower than the exposure rate in the workplace (63%) as reported in the GATS Bangladesh survey [10], but comparable to the exposure rate (45%) in public places [10]. The high SHS exposure rate in the workplace may be due to the fact that the smoking prevalence among Bangladeshi men is high and the majority of the workforces are men. Also, the partial workplace smoking restrictions implemented in 2005 are not well enforced [9].

Our findings showed that the SHS exposure prevalence at home was higher in females than males (48% versus 34%). This is consistent with findings in China [13], Korea [14] and Spain [15]. The high exposure prevalence among females is related to Bangladeshi social norms that disapprove of smoking by women; high smoking rates among males; and no or limited enforcement of household smoking bans [11]. In Bangladeshi culture, especially in rural areas, most women are housewives and spend most of their time at home, which increases their risk to SHS exposure from male smokers in the household. The dominant role of males in Bangladeshi society combined with respect to the household head (i.e. father) may also explain why father’s smoking status was a significant predictor for SHS exposure in the current study. To protect these vulnerable women from SHS exposure related morbidity and mortality, there is a need to empower women with information about SHS exposure related health effects and the measures they can take to avoid SHS exposure.

Consistent with findings in other countries [16-18], younger age was associated with SHS exposure among non-smokers. This might be due to the way families live in Bangladesh where three generations often live together in the same home. Because smoking prevalence among the older generation (aged 45–54: 26%; aged 55 or above: 22%) was higher than among young adults (aged 15–24:3%) [9], the young adult is more likely to live with current smokers than the older generation, resulting in more SHS exposure. Another factor may be the cultural tradition in Bangladesh of respecting one’s elders, which discourages arguing against elderly members’ smoking at home.

Many nonsmoking Bangladeshis in the study did not know that SHS causes lung cancer in non-smokers and not knowing was significantly associated with SHS exposure. While this finding is consistent with studies elsewhere [16,18], this underscores the need to increase non-smoking Bangladeshis’ knowledge of SHS exposure and its health risks. This low perception of risk might be related to the fact that many non-smoking Bangladeshis did not perceive themselves to be at risk from SHS exposure and did not take any protective measures to avoid SHS exposure. Health education campaigns [19,20] are critical to improve people’s knowledge of and attitudes towards SHS exposure.

Consistent with previous studies [18,20,21], our study showed that Bangladeshis with less education were more likely than more highly educated Bangladeshis to report SHS exposure. An earlier study reported that Bangladeshis with low socioeconomic status were more likely to be exposed to SHS [11]. This educational disparity in SHS exposure among Bangladeshis underscores the need for targeted educational interventions to improve health-related knowledge among the less educated Bangladeshis and emphasize the promotion of smoke-free home policies to this disadvantaged population group. Educating smokers about smoke-free homes would also increase smokers’ likelihood of quitting smoking and decrease cigarette consumption [22].

Somewhat unexpectedly, Bangladeshis with a moderate income (5000–10,000 taka) had higher SHS exposure at home. We have further explored this and found that there is a confounding effect with residence. In urban areas, SHS exposure decreased within increasing income (p <0.001). SHS exposure did not vary by income levels in the slum areas and in rural areas; SHS exposure was highest in the middle income group (data not shown). This phenomenon can be explained by the existence of wide variation of income among urban residents, but not among rural residents. The income inequality is less pronounced in rural areas and almost nonexistent in slum areas of Bangladesh.

The study has several limitations. First, the cross-sectional design of the study limits causal inferences about the findings. Second, SHS exposure was defined as a proxy measure constructed from self-reported home smoking rules and household composition of smokers, and might thus not always reflect real exposure to SHS at home. In addition, this measure does not provide information about dimensions of exposure such as frequency and intensity. While we recognize the limitations of the exposure variable, given the nature of the ITC survey, and given that no objective measures of SHS were employed during data collection, this measure still provides an indication of the possible exposure to SHS among non-smokers in Bangladesh. , Third, the current paper focused on household SHS exposure and did not identify other sources of exposure such as workplaces or outdoors. Therefore, the findings cannot be generalizable to SHS exposure outside of home. Fourth, there is a great gender imbalance in the study (74% participants were female). The gender imbalance in this study is an artifact of the sampling procedure and the low prevalence of smoking among Bangladeshi women. With respect to factors associated with SHS exposure, we controlled for gender in our regression model, so even after controlling for the gender imbalance, we still see significant predictors of exposure. Moreover, when we stratified the bivariate associations by gender, trends and significance tests are generally similar. So we think the gender imbalance is less of a problem for factors associated with SHS exposure, especially since we control for gender in the logistic regression model.Finally, data were collected by trained interviewers who followed written interviewer guidelines. Any difference between their understanding and explanation of the questions asked could result in bias in information collected. However, such bias was minimized by the periodical observation of interviews by the senior research team members and bi-weekly meetings. Despite these limitations, this study supports the need to develop comprehensive interventions to promote SHS exposure reduction at home.

## Conclusion

This study documents that almost half of non-smoking Bangladeshi adults are exposed to SHS in the home. Many of the predictors of SHS exposure identified in the current study are amenable to change. Policy strategies should consider these factors in the design of interventions addressing SHS exposure reduction among the public. Both population-based policies and clinical encounters are needed to improve public understanding about the health hazards of SHS exposure and to encourage the public in enacting smoking policies that promote a smoke-free home. It is also suggestive that smoke-free indoor policies should not only be enforced among non-smokers (mostly female) given the dominant role of males in Bangladeshi society. Therefore, attention should be also or especially given to (male) smokers in enforcing smoke-free indoor policies. There is evidence that smoke-free indoor policies are effective and easy to implement for the prevention of exposure to SHS, and a 100% ban on smoking in any indoor environments (i.e. at home) is the only intervention that can effectively protect people from the hazards of SHS exposure. Therefore, there is a need to implement evidence-based tobacco control strategies to promote SHS exposure reduction in the home. Interventions should deliver targeted messages to reach those in the low socioeconomic status group.

## Abbreviations

FCTC: Framework Convention on Tobacco Control; GATS: Global Adult Tobacco Survey; ITC: International Tobacco Control Policy Evaluation Project; TSP: Tobacco smoke pollution; WHO: World Health Organization.

## Competing interests

The authors declare that they have no competing interests.

## Authors’ contributions

GTF, NN, ASA planned the study. ACKQ coordinated the overall implementation of the project. NN and AGH directed the project implementation and field work in Bangladesh. ASA took the lead to draft the paper. PD conducted all the analyses and gave critical comments on the initial drafts. GS helped in the literature review process and provided critical input in the first draft of the manuscript. All authors approved the final draft of the paper.

## Pre-publication history

The pre-publication history for this paper can be accessed here:

http://www.biomedcentral.com/1471-2466/14/117/prepub
